# Yeast associated with flower longicorn beetle *Leptura ochraceofasciata* (Cerambycidae: Lepturinae), with implication for its function in symbiosis

**DOI:** 10.1371/journal.pone.0282351

**Published:** 2023-03-22

**Authors:** Mako Kishigami, Fumiaki Matsuoka, Akiteru Maeno, Shohei Yamagishi, Hisashi Abe, Wataru Toki

**Affiliations:** 1 Graduate School of Bioagricultural Sciences, Nagoya University, Nagoya, Aichi, Japan; 2 National Institute of Genetics, Mishima, Shizuoka, Japan; 3 Forestry and Forest Research Institute, Tsukuba, Ibaraki, Japan; Academia Sinica, TAIWAN

## Abstract

Wood is difficult for most animals to digest due to large amounts of indigestible polymers, but some wood-feeding insects are considered to be able to utilize it as food with the aid of microbial symbionts. Most members of flower longicorn beetles (Coleoptera: Cerambycidae: Lepturinae) feed on nectar and pollen of flowers as adults and wood as larvae. In some lepturines, associations with yeasts are known: female adults possess fungus-storing organs (termed mycetangia) at ovipositors, and larvae also possess such organs (termed mycetomes) in their midguts to carry the associated yeasts. Despite the high diversity of Lepturinae in the world, lepturine-yeast associations, such as the consistency of associated yeasts among the beetle’s developmental stages and ecological function of yeast symbionts, have been poorly documented. Here, we investigated the yeast symbiont of the Japanese common lepturine *Leptura ochraceofasciata*. X-ray computed microtomography revealed that a pair of tube-like, S-shaped mycetangia was located at the basal part of the ovipositor and that a muscle bundle joined the apex of the mycetangium to spiculum ventrale of sternum VIII. All female adults harbored only one yeast species, *Scheffersomyces insectosa*, in the mycetangia. All larvae harbored *S*. *insectosa* exclusively in the mycetomes. *Scheffersomyces insectosa* was also recovered from surfaces of eggs. *Scheffersomyces insectosa* assimilated wood-associated sugars including xylose, cellobiose, and xylan in culture. These results suggest the intimate association between *L*. *ochraceofasciata* and *S*. *insectosa*: *S*. *insectosa* is transmitted from the mother to offspring during oviposition and may be related to larval growth in wood.

## Introduction

Wood is composed of indigestible polymers, such as cellulose, hemicelluloses, and lignin, and thus wood is unavailable for most animals as food [1]. In nature, however, diverse insects inhabit woody materials. Studies have suggested that microbial symbionts enable wood-inhabiting insects to utilize wood. For example, fungal symbionts of some ambrosia beetles (Curculionidae: Scolytinae and Platypodinae) contribute to degrading wood [2, 3]. Xylophagous (wood-feeding) beetles such as passalid beetles (Passalidae), stag beetles (Lucanidae), and longicorn beetles (Cerambycidae) are associated with xylose-fermenting yeasts [4–6]. Yeasts originating from ship-timber beetles (Lymexylidae) are capable of assimilating wood-associated sugars [7]. Interestingly, some of these insects have evolved specialized pocket-like organs called mycetangia or mycangia for transmitting symbiotic microorganisms from the mother to offspring [8–10]. In most wood-inhabiting insects, however, insect-microbe associations have been poorly documented.

Recently, the morphology of symbiont-carrying organs of insects has been observed in a non-destructive way using X-ray computed microtomography (micro-CT), including mycetangia in ambrosia beetles (e.g., [11, 12]) and the hemipteran bacteriome (bacteria-carrying organ) [13]. This technique can produce detailed 3D images of microstructures located inside the body, which contributes to a better understanding of insect-microbe associations. In other mycetangia-bearing beetles, however, mycetangia have not been examined by micro-CT.

**Fig 1:**
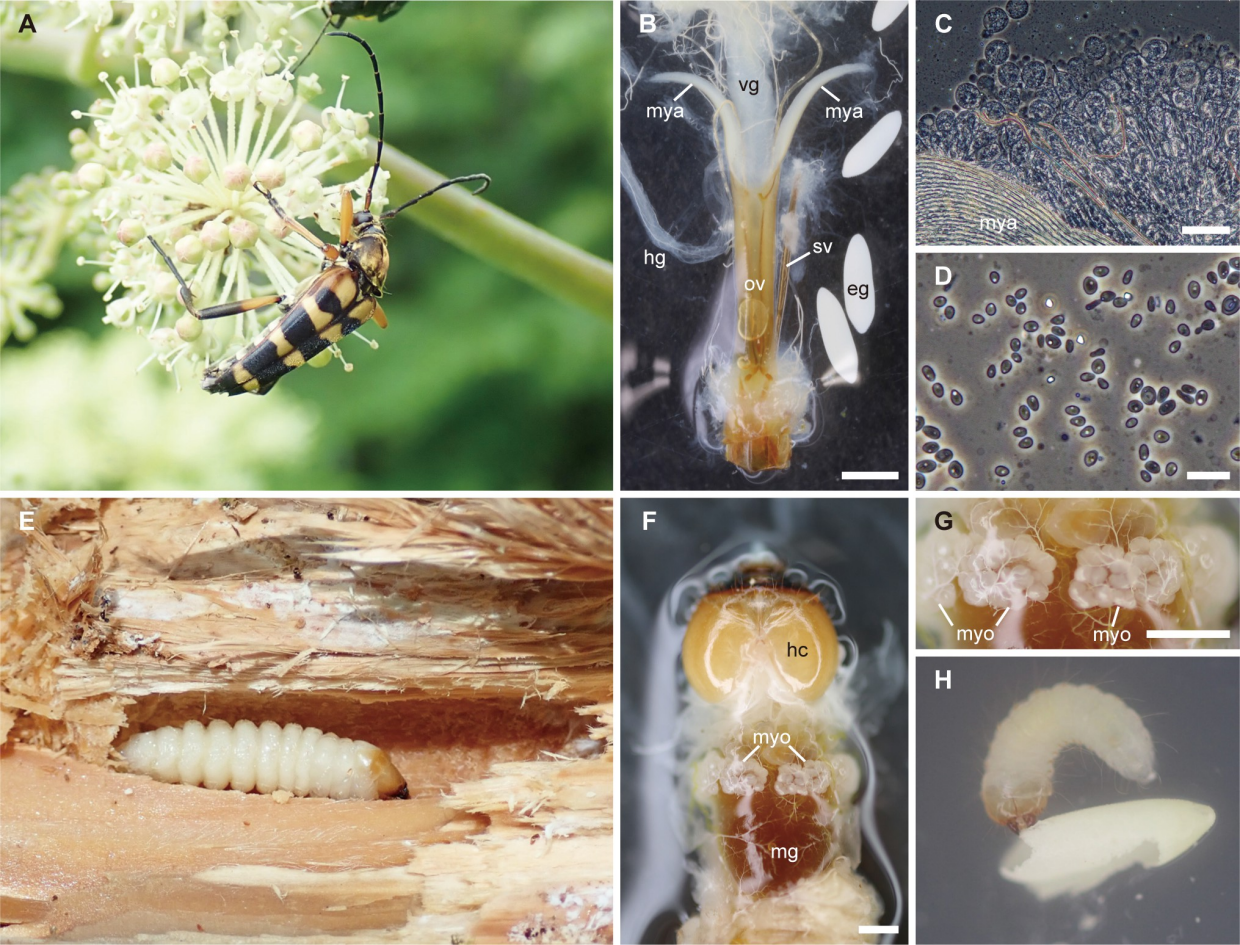
The flower longicorn beetle *Leptura ochraceofasciata* and associated fungi. (A) A male adult of *L*. *ochraceofasciata* visiting flowers of *Aralia cordata*; (B) ovipositor, dorsal view; (C) contents of a mycetangium; (D) yeasts in a mycetangium; (E) a larva in decayed wood of *Chamaecyparis obtusa*; (F) a larval gut, dorsal view; (G) magnification of mycetomes; (H) a hatched larva feeding on its eggshell. Abbreviations: eg, egg; hc, head capsule; hg, hindgut; mg, midgut; mya, mycetangium; myo, mycetome; ov, ovipositor; sv, spiculum ventrale; vg, vagina. Scale bars = 1 mm (B, F, G), 50 μm (C), 10 μm (D).

The flower longicorn beetle (Cerambycidae: Lepturinae) consists of more than 1500 species in 210 genera in the world [14]. Adults of most lepturines feed on pollen and nectar of flowers and the larvae feed on decayed wood [14, 15]. Associations between lepturine beetles and yeasts have been suggested [5, 16]. Female adults of lepturines have a pair of mycetangia, termed intersegmental tubules by Buchner (1965) [16], at the basal part of ovipositors, and species with long mycetangia harbor yeasts in mycetangia [17] (Fig 1B). The larvae possess a specialized organ termed the mycetome containing yeasts at the basal part of the midgut [5, 16] (Fig 1F and 1G). Yeasts are constantly expelled from mycetomes into the digestive tract in *Rhagium inquisitor* (Linnaeus) and *Stictoleptura rubra* (Linnaeus) [5]. It is considered that vertical transmission of yeast symbionts occurs through the surface of eggshells [16].

Despite the marked diversity and worldwide distribution of Lepturinae, understanding of the insect-yeast association is limited to a small number of European and American species [5, 16]. Consistency of associated yeasts between the adult and larva has only been revealed in *R*. *inquisitor*-*Hyphopichia rhagii* and *R*. *mordax* (DeGeer)-*Scheffersomyces* sp. associations [5, 18–20]. The enzymatic ability of lepturine-associated yeast symbionts to digest wood-derived materials has been poorly documented [18].

The lepturine beetle, *Leptura ochraceofasciata* (Motschulsky) is distributed in East Asia [21]. In Japan, *L*. *ochraceofasciata* is one of the most common lepturines and divided into three subspecies [21, 22]. The adults are abundant on flowers in summer and the larvae feed on dead wood of various families of broadleaved and coniferous trees [23] (Fig 1A and 1E). To date, its association with yeasts has not been investigated. If *L*. *ochraceofasciata* is closely associated with a specific yeast, it is expected that the mycetangia of female adults would contain the same yeast species among individuals. If the associated yeast is transmitted vertically through the egg surface like in other lepturines, it may be isolated from both mycetomes of larvae and surfaces of eggs.

In the present study, we aimed to determine whether *L*. *ochraceofasciata* has an association with a specific yeast and clarify the assimilating abilities of the yeast. We observed detailed morphology of mycetangia using micro-CT techniques and conducted microbial isolation from the mycetangia of female adults, mycetomes of larvae, and surfaces of eggs and a carbon assimilation test for the yeast isolated. Finally, the association between the lepturine insect and yeast is discussed.

## Materials and methods

### Insects

For yeast isolation, 11 female adults of *L*. *o*. *ochraceofasciata* were collected in central Honshu, Japan in July and August 2018, July 2020, and June 2021 (Table 1). Of those, eight were collected when visiting flowers of *Angelica pubescens* (Apiaceae), *Cynanchum caudatum* (Apocynaceae), and *Hydrangea paniculata* (Hydrangeaceae). One (individual ID: Fi10) was captured when she just emerged from decayed wood of *Chamaecyparis obtusa* (Cupressaceae). Two (Fs1 and Fs2) originated from a piece of decayed wood of *Abies* sp. (Pinaceae), which was identified by microscopic observation following the procedure of Iimura et al. (2021) [24]. The sampled wood was placed in the laboratory at room temperature. One female (Fs1) emerged from the wood and the other (Fs2) was in the pupal chamber when the wood was split using a wood-cutting knife. In addition, a female adult (Fy1) of *L*. *o*. *ochrotela* Bates was collected on a fallen dead tree (unidentified conifer) in Kyushu, Japan on 22 July 2020 (Table 1). Water or honey solution was added to keep these adult samples alive until use.

**Table 1:** Adults, larvae, and eggs of *Leptura ochraceofasciata* used for yeast isolation.

Taxon	No. of individuals (ID)	Collection date	Locality	Collecting situation
*L*. *o*. *ochraceofasciata*	3 female adults (Fi1-Fi3)	19 July, 2018	Inabu, Toyota City, Aichi Prefecture, Japan (35°13‘N, 137°34‘E, 1000-m alt.)	Visiting flowers of *Hydrangea paniculata*
	1 female adult (Fi10)	16 July, 2020	Inabu, Toyota City, Aichi Prefecture, Japan (35°13‘N, 137°34‘E, 1000-m alt.)	Emerged from decayed wood of *Chamaecyparis obtusa* (16 July, 2020)
	1 female adult (Fi8)	19 July, 2020	Mt. Dando, Shitara Town, Aichi Prefecture, Japan (35°07‘N, 137°28‘E, 900-m alt.)	Visiting flowers of *H*. *paniculata*
	1 female adult (Fo1)	1 Aug., 2018	Kaida Highlands, Kiso Town, Nagano Prefecture, Japan (35°56‘N, 137°35‘E, 1200-m alt.)	Visiting flowers of *Angelica pubescens*
	1 female adult (Fo2)	1 Aug., 2018	Kaida Highlands, Kiso Town, Nagano Prefecture, Japan (35°56‘N, 137°35‘E, 1200-m alt.)	Visiting flowers of *H*. *paniculata*
	1 female adult (Fo3)	1 Aug., 2018	Hiwada Highlands, Takayama City, Gifu Prefecture, Japan (35°57‘N, 137°29‘E, 1600-m alt.)	Visiting flowers of *H*. *paniculata*
	1 female adult (Fk1)	22 July, 2020	Azusayama, Kawakami Village, Nagano Prefecture, Japan (35°58‘N, 138°42‘E, 1380-m alt.)	Visiting flowers of *Cynanchum caudatum*
	1 female adult (Fs1)	10 June, 2021	Ookawara, Ooshika Village, Nagano Prefecture, Japan (35°33‘N, 138°05‘E, 1740-m alt.)	Emerged from decayed wood of *Abies* sp. (2 July, 2021)
	1 female adult (Fs2)	10 June, 2021	Ookawara, Ooshika Village, Nagano Prefecture, Japan (35°33‘N, 138°05‘E, 1740-m alt.)	In a pupal chamber in decayed wood of *Abies* sp. (2 July, 2021)
	5 larvae (Li1-Li5)	3–26 June, 2020	Inabu, Toyota City, Aichi Prefecture, Japan (35°13‘N, 137°34‘E, 1000-m alt.)	Inside decayed wood of *Ch*. *obtusa*
	3 eggs (E1-E3)	30 July, 2020^a^	Inabu, Toyota City, Aichi Prefecture, Japan (35°13‘N, 137°34‘E, 1000-m alt.)	Oviposited in the laboratory (5 Aug., 2020)
*L*. *o*. *ochrotela*	1 female adult (Fy1)	22 July, 2020	Mt. Kirishima, Kirishima City, Kagoshima Prefecture, Japan (31°54‘N, 130°51‘E, 900-m alt.)	Visiting a fallen dead tree (unidentified conifer)

^a^Ten female adults captured on flowers of *H*. *paniculata* were used to obtain eggs.

To observe mycetangia of adults, a female of *L*. *o*. *ochraceofasciata* was obtained from flowers of *H*. *paniculata* on Mt. Dando, Shitara Town, Aichi Prefecture, Japan (35°07‘N, 137°28‘E, 900-m altitude) on 16 July, 2021 and two females of *L*. *o*. *ochraceofasciata* were obtained from flowers of *Fallopia japonica* (Polygonaceae) in Nigorigo, Gero City, Gifu Prefecture, Japan (35°55‘N, 137°27‘E, 1740-m altitude) on 30 July, 2022.

Five lepturine larvae were collected from rotten wood of *Ch*. *obtusa* in Inabu, Toyota City, Aichi Prefecture, Japan (35°13‘N, 137°34‘E, 1000-m altitude) on 3 to 26 June, 2020 (Table 1). They were individually stored in plastic tubes at 10 to 15°C.

Samples were weighed using a digital scale when dissected. Body and elytral lengths of adults were also determined using digital calipers.

To obtain eggs, ten adult females of *L*. *o*. *ochraceofasciata* (elytral length: mean ± SD = 11.63 ± 0.85 mm, *n* = 10) were captured in Inabu on 30 July, 2020. As an oviposition substrate, a rolled corkboard (4-cm diameter × 6 cm) with a piece of decayed wood of *Ch*. *obtusa* where larvae of *L*. *o*. *ochraceofasciata* were found in Inabu placed at the core was put in a plastic container (10 × 10 × 10 cm). Then, adult females were placed in the container and allowed to lay eggs at room temperature (ca. 25°C) under florescent light. Twenty-six arbitrarily selected eggs were individually placed onto a Petri dish (3-cm diameter) with a piece of moistened paper and incubated at 25°C in the dark. For yeast isolation, three 14-day-reared eggs were selected arbitrarily. The other eggs were incubated until hatching to observe behaviors of hatched larvae.

To identify larval samples, we applied a molecular approach. Adults of 13 species from 9 genera in the tribe Lepturini and 2 species from 2 genera in the tribe Rhagiini used as an outgroup were collected in Japan (Table 2). Note that all species of the genus *Leptura* recorded in Aichi Prefecture were used for the analysis except for an uncommon species *L*. *kusamai* Ohbayashi et Nakane [25]. The captured samples were preserved in absolute ethanol.

**Table 2:** Samples of adult lepturines used for larval identification.

Species	Collection date	Locality	Accession no. for COI
Tribe Lepturini (ingroup)			
*Anoploderomorpha excavata* (Bates)	31 July, 2020	Hakkaisan, Otaki Village, Nagano Prefecture, Japan (35°52‘N, 137°32‘E, 1670-m alt.)	LC733218
*Japanostrangalia dentatipennis* (Pic)	6 Aug., 2020	Hiwada Highlands, Takayama City, Gifu Prefecture, Japan (35°57‘N, 137°29‘E, 1600-m alt.)	LC733219
*Leptura annularis* Fabricius	31 July, 2020	Hakkaisan, Otaki Village, Nagano Prefecture, Japan (35°52‘N, 137°32‘E, 1670-m alt.)	LC733220
*Leptura dimorpha* Bates	26 July, 2019	Sengendaru Highlands, Takayama City, Gifu Prefecture, Japan (35°57‘N, 137°29‘E, ca. 1800-m alt.)	LC733221
*Leptura latipennis* (Matsushita)	10 June, 2022	Azusayama, Kawakami Village, Nagano Prefecture, Japan (35°58‘N, 138°42‘E, 1380-m alt.)	LC733222
*Leptura ochraceofasciata ochraceofasciata* (Motschulsky)	4 Aug., 2020	Shirabiso Highlands, Iida City, Nagano Prefecture, Japan (35°26‘N, 138°01‘E, ca. 1900-m alt.)	LC733223
*Leptura subtilis* Bates	30 July, 2021	Inabu, Toyota City, Aichi Prefecture, Japan (35°13‘N, 137°34‘E, 1000-m alt.)	LC733224
*Macroleptura regalis* (Bates)	9 Aug., 2019	Inabu, Toyota City, Aichi Prefecture, Japan (35°13‘N, 137°34‘E, 1000-m alt.)	LC733225
*Pachytodes cometes* (Bates)	16 July, 2020	Inabu, Toyota City, Aichi Prefecture, Japan (35°13‘N, 137°34‘E, 1000-m alt.)	LC733226
*Parastrangalis nymphula* (Bates)	17 July, 2020	Azusayama, Kawakami Village, Nagano Prefecture, Japan (35°58‘N, 138°42‘E, 1380-m alt.)	LC733227
*Pedostrangalia femoralis* (Motschulsky)	10 June, 2022	Azusayama, Kawakami Village, Nagano Prefecture, Japan (35°58‘N, 138°42‘E, 1380-m alt.)	LC733228
*Strangalia koyaensis* Matsushita	16 July, 2020	Inabu, Toyota City, Aichi Prefecture, Japan (35°13‘N, 137°34‘E, 1000-m alt.)	LC733229
*Strangalomorpha tenuis* Solsky	31 July, 2018	Hakkaisan, Otaki Village, Nagano Prefecture, Japan (35°52‘N, 137°32‘E, 1670-m alt.)	LC733230
Tribe Rhagiini (outgroup)			
*Lemula decipiens* Bates	25 June, 2019	Yanagiran Pass, Takayama City, Gifu Prefecture, Japan (35°57‘N, 137°29‘E, ca. 1700-m alt.)	LC733231
*Paragaurotes doris* (Bates)	31 July, 2020	Hakkaisan, Otaki Village, Nagano Prefecture, Japan (35°52‘N, 137°32‘E, 1670-m alt.)	LC733232

No specific permits were required for the described field studies. The locations are not privately owned or protected in any way. The field studies did not involve protected species. All applicable international, national, and/or institutional guidelines for the care and use of animals were followed.

### Fungus-storing organs of adults and larvae

Living adults and larvae were dissected using fire-sterilized tweezers under a stereo-microscope. The presence or absence of the mycetangia in adults and mycetomes in larvae were recorded. Images of them were photographed using an EOS Kiss X8i digital camera (Canon, Tokyo, Japan). The length of either the left or right mycetangium was measured using ImageJ 1.47t [26] for female adults used for yeast isolation except for Fs1 and Fs2, and the relative mycetangial length was calculated as the mycetangial length/elytral length. These mycetangia and mycetomes were further used for yeast isolation.

In addition, living adults from Nigorigo were dissected, the mycetangia were removed using fire-sterilized tweezers, and their contents were observed by microscopy.

### Micro-CT observation of fungus-storing organs of adults

Antennae and legs of a living female adult (elytral length: 12.04 mm, weight: 181.2 mg) obtained from Dando were cut using tweezers. Then, the body was fixed with Carnoy solution for 2 days at room temperature, stained with 25% Lugol’s solution for 4 days at room temperature, and embedded in 0.5% agarose gel [27].

To observe the mycetangial structure, first, we scanned the abdomen without dissection. Because aggregation of membranous organs around the base of the ovipositor made it difficult to distinguish mycetangia, we carefully removed eggs, ovaries, the digestive tract, and spiculum ventrale of sternum VIII using tweezers under a stereo-microscope. The dissected genitalia was restained with 25% Lugol’s solution for 1 day at room temperature, embedded with 0.5% agarose gel, and scanned. As shapes and positions of mycetangia were not different between before and after dissection, scanned data of dissected genitalia were used for analyses.

The dissected genitalia in a stand-mounted 200-μm micropipette tip (BM Equipment, Tokyo, Japan) was scanned using the ScanXmate-E090S105 (ComscanTechno, Kanagawa, Japan). The genitalia was rotated 360° in steps of 0.24°, generating 1500 projection images of 992 × 992 pixels. The X-ray source was set at 85 kV and 90 μA. The scan data were reconstructed at an isotropic resolution of 2.8 μm, and converted into a tiff image dataset using coneCTexpress software (ComscanTechno). Digital cross-sections and 3D models were made using OsiriX MD (Pixmeo, Bernex, Switzerland) and Imaris v9.8 (Bitplane, Zurich, Switzerland). Finally, supplemental movies were edited using Adobe Premiere Pro (Adobe, San Jose, CA, USA) (S1 and S2 Movies).

### Yeast isolation from adults, larvae, and eggs

For adults, a mycetangium was removed from the ovipositor using fire-sterilized tweezers, surface-washed with sterile water for 10 s twice, and placed in a 2-mL tube containing 1000 μL of sterile water. It was cut into small pieces using a fire-sterilized injection needle and vortexed vigorously.

For larvae, mycetomes were removed from the guts, surface-washed with sterile water for 10 s three times, and placed in a 1.5-mL tube containing 500 or 1000 μL of sterile water. They were ground using an autoclaved pestle and vortexed vigorously.

For eggs, each of them was directly placed in a 2-mL tube containing 1000 μL of sterile water, and vortexed vigorously.

Then, 50 μL of the suspension was spread over potato dextrose agar (PDA) (Difco, Detroit, MI, USA) plates (9-cm diameter) containing 20 μg/mL of rifampicin (FUJIFILM Wako Pure Chemical, Osaka, Japan). Three replicates were made for each of the samples from adults and larvae, and one for each of the egg samples. The plates were incubated at 25°C in the dark until yeast colonies appeared. The fungal colonies that grew on the plates were roughly classified based on their morphological traits (morphotype), and the number of colonies of each morphotype (colony forming units = CFU) was counted. When too many colonies were present on the plates, 10- and 100-fold dilutions were subsequently made using the original suspension stored at 5°C, and the above-mentioned microbial isolation was reconducted using the diluted solutions. Note that the CFU values were not comparable among samples due to variable storing periods (0 to 92 days) of suspension before use. Four or eight colonies per morphotype were selected arbitrarily for subsequent culturing and DNA analysis.

### DNA sequencing analysis of yeasts

To identify yeasts isolated from *L*. *ochraceofasciata*, DNA sequences (ca. 600 bps) in the D1/D2 domain of the 26S rRNA (26S) gene were determined. In addition, to estimate their phylogenetic positions, DNA sequences of the internal transcribed spacer region and 5.8S rRNA (ITS/5.8S) gene (ca. 600 bps) and those of the translation elongation factor-1α (TEF) gene (ca. 800 bps) were determined for the representative isolates.

For DNA extraction, small pellets of the yeast colonies were suspended in 100 μL of TE buffer and incubated at 99°C for 10 min. After centrifugation, 30 μL of the supernatant containing DNA was stored and directly used for polymerase chain reaction (PCR) amplification. The following primer pairs were used for PCR: LS1 (5’-AGTACCCGCTGAACTTAAG-3’) (forward) [28] and NL4 (5’-GGTCCGTGTTTCAAGACGG-3’) (reverse) [29] for 26S, ITS-5 (5’-GGAAGTAAAAGTCGTAACAAGG-3’) and ITS-4 (5’-TCCTCCGCTTATTGATATGC-3’) [30] for ITS/5.8S, and YTEF-1G (5’-GGTAAGGGTTCTTTCAAGTACGCTTGGG-3’) (forward) and YTEF-6G (5’-CGTTCTTGGAGTCACCACAGACGTTACCTC-3’) (reverse) [31] for TEF.

The PCR products were purified using Exo SAP-IT (Thermo Fisher Scientific, Waltham, MA, USA), and directly sequenced using BigDye Terminators (Thermo Fisher Scientific) and ABI PRISM 3130xl, 3730xl Genetic Analyzer (Thermo Fisher Scientific). Nucleotide sequence data reported in this study have been deposited in the DNA Data Bank of Japan (DDBJ) with accession numbers LC732211–LC732272 (see Table 3). The sequences were subjected to BLASTn searches for identification [32]. For *Scheffersomyces* yeasts, multiple alignments of the nucleotide sequences were generated using the program ClustalW in MEGA X [33]. Molecular phylogenetic analysis was conducted by the neighbor-joining method using MEGA X with 1000 bootstrap replicates.

**Table 3:** DDBJ/EMBL/GenBank accession numbers for each *Leptura ochraceofasciata*-related yeast strain sequenced.

Strain	Estimated taxon	Accession no.			Maximum partial similarity (%)		
		26S	ITS/5.8S	TEF	26S	ITS/5.8S	TEF
Fi1-1-1	*Scheffersomyces insectosa*	LC732211	LC732233	LC732253	100	100	99.5
Fi2-1-1	*Scheffersomyces insectosa*	LC732212	LC732234	LC732254	100	100	99.5
Fi3-1-1	*Scheffersomyces insectosa*	LC732213	LC732235	LC732255	100	100	99.7
Fi8-1-1	*Scheffersomyces insectosa*	LC732214	LC732236	LC732256	100	100	99.5
Fi10-1-1	*Scheffersomyces insectosa*	LC732215	LC732237	LC732257	100	100	99.7
Fo1-1-1	*Scheffersomyces insectosa*	LC732216	LC732238	LC732258	100	99.8	99.2
Fo2-1-1	*Scheffersomyces insectosa*	LC732217	LC732239	LC732259	100	99.8	99.2
Fo3-1-1	*Scheffersomyces insectosa*	LC732218	LC732240	LC732260	100	99.8	99.2
Fy1-1-1	*Scheffersomyces insectosa*	LC732219	LC732241	LC732261	100	100	99.7
Fk1-1-1	*Scheffersomyces insectosa*	LC732220	LC732242	LC732262	100	100	99.2
Fs1-1	*Scheffersomyces insectosa*	LC732221	LC732243	LC732263	100	99.8	99.7
Fs2-1	*Scheffersomyces insectosa*	LC732222	LC732244	LC732264	100	100	99.7
Li1-1	*Scheffersomyces insectosa*	LC732223	LC732245	LC732265	100	100	99.7
Li2-1	*Scheffersomyces insectosa*	LC732224	LC732246	LC732266	100	100	99.4
Li3-1	*Scheffersomyces insectosa*	LC732225	LC732247	LC732267	100	100	99.5
Li4-1	*Scheffersomyces insectosa*	LC732226	LC732248	LC732268	100	100	99.4
Li5-1	*Scheffersomyces insectosa*	LC732227	LC732249	LC732269	100	100	99.5
E1-1-1	*Scheffersomyces insectosa*	LC732228	LC732250	LC732270	100	100	99.7
E2-1-1	*Scheffersomyces insectosa*	LC732229	LC732251	LC732271	100	100	99.7
E3-1-1	*Scheffersomyces insectosa*	LC732230	LC732252	LC732272	100	100	99.7
E1-1-2	*Meyerozyma* sp.	LC732231	n.a.	n.a.	100	n.a.	n.a.
E3-1-3	*Meyerozyma* sp.	LC732232	n.a.	n.a.	100	n.a.	n.a.

n.a., not applicable.

### Identification of larvae

To identify larvae collected in this study, DNA sequences in the mitochondrial cytochrome oxidase subunit I (COI) gene (658 bps) were determined. DNA was extracted from muscle tissues of adults and heads of larvae using PrepMan Ultra Reagent (Life Technologies, Warrington, UK). The following primer pair was used for PCR: LCO1490 (5’-GGTCAACAAATCATAAAGATATTGG-3’) (forward) and HCO2198 (5’-TAAACTTCAGGGTGACCAAAAAATCA-3’) (reverse) [34]. Purification of PCR products and sequencing were conducted in the above-mentioned manners (accession numbers: LC733218–LC733232 for adults, LC733233–LC733237 for larvae) (see Table 2). Then, the DNA sequences of larvae were compared with those of adults. A neighbor-joining phylogenetic tree was constructed using MEGA X with 1000 bootstrap replicates.

### Carbon assimilation test

The representative isolate (strain name: Fo1-1-1) of the yeast deriving from a female (Fo1) originating from the Kaida Highlands was cultured aerobically in 20 mL of yeast nitrogen base (YNB) (Difco) containing 0.5% glucose at 25°C in the dark for 2 to 3 days with shaking at 85 rpm. The culture media were centrifuged and cell pellets were suspended in sterile water.

To investigate the relationship between CFU and turbidity of the yeast examined, five types of yeast suspensions (OD_600_ = 0.05, 0.10, 0.50, 1.00, 1.25) were made. For each type of suspension, 50 μL of each dilution (i.e., 1/10^2^, 1/10^3^, 1/10^4^ equivalent of the original suspension) was spread onto a PDA plate (9-cm diameter) containing 20 μg/mL of rifampicin (3 replicates). The plates were incubated at 25°C in the dark for 2 days and the CFUs were counted.

For the carbon assimilation test, we made a suspension in which OD_600_ was adjusted to 0.10–0.12. Then, 50 μL of the cell suspension was added into a tube (2 mL) with 1 mL of each of 16 different media containing YNB and the following carbon sources: d-glucose (FUJIFILM Wako Pure Chemical), d-galactose (FUJIFILM Wako Pure Chemical), d-mannose (FUJIFILM Wako Pure Chemical), d-xylose (FUJIFILM Wako Pure Chemical), d-fructose (FUJIFILM Wako Pure Chemical), l-arabinose (FUJIFILM Wako Pure Chemical), l-rhamnose (Nacalai Tesque, Kyoto, Japan), d-glucuronic acid (FUJIFILM Wako Pure Chemical), d-galacturonic acid (FUJIFILM Wako Pure Chemical), cellobiose (FUJIFILM Wako Pure Chemical), sucrose (FUJIFILM Wako Pure Chemical), xylan from corn (Tokyo Chemical Industry, Tokyo, Japan), xylan from beech (SERVA Electrophoresis, Heidelberg, Germany), glucomannan from Konjac (Natural Life, Tokyo, Japan), carboxymethyl cellulose (FUJIFILM Wako Pure Chemical), and no carbon source (*n* = 3). The concentration of each carbon source was 0.5 g/L, except for xylan, at 1.5 g/L. As xylan from beech is insoluble, a high concentration of beech-xylan was used. To determine whether the assimilating ability is different between beech- and corn-xylans, the concentration of corn-xylan was adjusted to 1.5 g/L. The tubes were shaken at 85 rpm and incubated at 25°C in the dark for 7 days. Afterwards, OD_600_ was recorded to determine the growth of each strain. The degree of assimilation was scored according to the difference in the turbidity increase (ΔOD_600_) between culture media containing no and a given carbon source as follows: no growth (ΔOD_600_ < 0.03), weak growth (0.03 ≤ ΔOD_600_ < 0.10), moderate growth (0.10 ≤ ΔOD_600_ < 0.40), strong growth (0.40 ≤ ΔOD_600_ < 1.00), and very strong growth (1.00 ≤ ΔOD_600_) [7].

### Statistical analysis

Pearson’s correlation coefficient and the ordinary least squares (OLS) method were used to determine the relationship between two variables. Calculations were performed using R 3.5.1 [35].

## Results

### Fungus-storing organs of adults

All female adults of *L*. *o*. *ochraceofasciata* and *L*. *o*. *ochrotela* (body length: mean ± SD = 17.70 ± 1.19 mm, range = 16.20 to 19.98 mm, *n* = 12; elytral length: 12.32 ± 0.82 mm, range = 10.46 to 13.41 mm, *n* = 12; weight: 192.6 ± 37.8 mg, range = 115.2 to 247.7 mg, *n* = 12) had a pair of membranous, symmetrical, tube-like mycetangia (length: 2.85 ± 0.17 mm, range = 2.57 to 3.09 mm, *n* = 10; relative mycetangial length: 0.23 ± 0.02, range = 0.21 to 0.27, *n* = 10) at the base of the ovipositor (Fig 1B, S1 Table). The blind end of each mycetangium and anterior end of the spiculum ventrale of sternum VIII were connected by thin muscle tissues as reported in other species [16, 17] (Fig 1B). Note that we did not record the presence/absence of a secretion gland open to the mycetangia. When a mycetangium was removed from the ovipositor, a whitish fluid came out. In a mycetangium, cysts containing large numbers of yeast cells were abundant (*n* = 2) (Fig 1C and 1D). Some of these yeast cells were budding (Fig 1D).

The mycetangial length and relative mycetangial length were not correlated with the elytral length (*r* = 0.00, *P* = 1.000, *n* = 10 for mycetangial length; *r* = –0.53, *P* = 0.123, *n* = 10 for relative mycetangial length). This was also the case for body weight (*r* = 0.11, *P* = 0.764, *n* = 10 for mycetangial length; *r* = –0.45, *P* = 0.191, *n* = 10 for relative mycetangial length).

### Micro-CT observation of fungus-storing organs of adults

**Fig 2:**
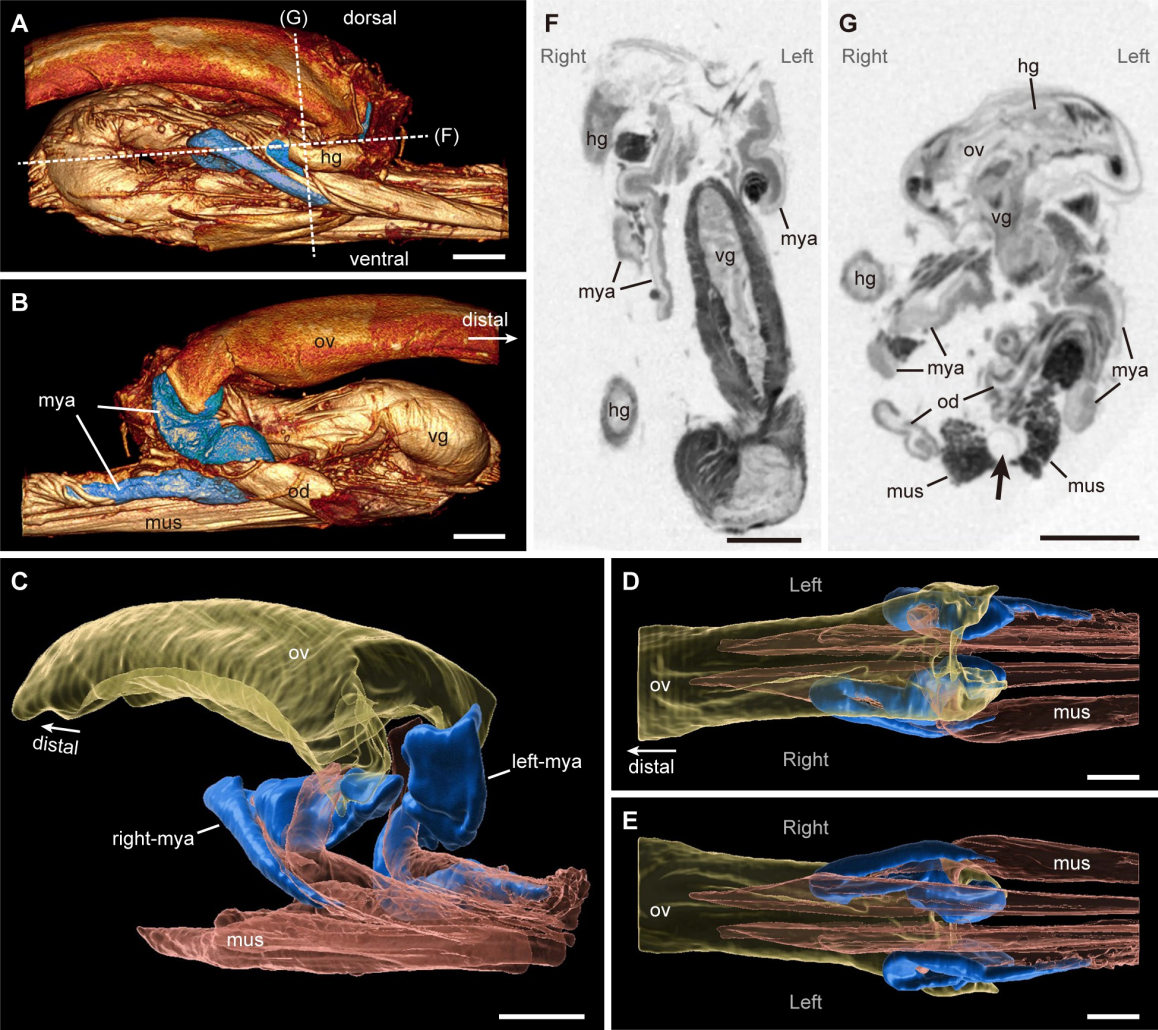
Micro-computed tomography (CT) of a dissected female genitalia of *Leptura ochraceofasciata*. (A–E) 3D reconstruction of female genitalia including mycetangia (blue) based on micro-CT, right lateral (A), left lateral (B), right diagonal (C), dorsal (D), and ventral (E) views. Mycetangia, the ovipositor (yellow), and musculature (red) along the spiculum ventrale of sternum VIII are shown in (C–E). (F, G) Single micro-CT sections of female genitalia from dashed lines in (A), ventral (F) and anterior (G) views. Note that some organs including the spiculum ventrale of sternum VIII and parts of the oviducts and hindgut were removed prior to scanning. A part of hindgut image was also removed for improving the visuality of mycetangia in (A). A black arrow indicates the original position of the spiculum ventrale of sternum VIII in (G). Abbreviations: hg, hindgut; mus, musculature along spiculum ventrale of sternum VIII; mya, mycetangium; od, lateral oviduct; ov, ovipositor; vg, vagina. Scale bars = 300 μm.

Micro-CT revealed that paired tube-like mycetangia were located at the basal part of the ovipositor (Fig 2, S1 and S2 Movies), as observed by dissection under a stereo-microscope (Fig 1B). Each mycetangium was S-shaped: the basal part curved posteriorly and ventrally, the mid-part was located between the lateral oviduct and vagina and curved anteriorly with surrounding the lateral oviduct, and the blind end connected with muscle tissues along the spiculum ventrale of sternum VIII (Fig 2, S2 Movie). The 3D images of the left and right mycetangia were asymmetrical in shape and position within the body (Fig 2C–2E, S2 Movie).

### Identification of larvae

The DNA sequences (658 bps) for the COI gene were 99.5–100% identical to each other among five larvae and closest to that of the *L*. *o*. *ochraceofasciata* adult (99.7–99.8% identity).

**Fig 3:**
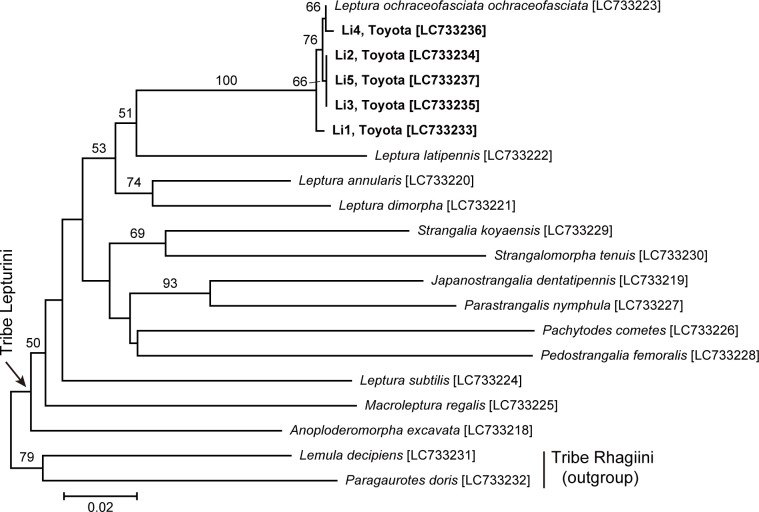
A neighbor-joining phylogenetic tree of Lepturinae constructed using DNA sequences of the COI gene (532 bps). Bootstrap values (1000 replicates) of 50% or higher are shown at the nodes. Sequence accession numbers are presented in brackets. For larval specimens, the individual ID and collection locality are indicated in bold.

Due to multiple alignments of the DNA sequences of the sampled larvae and lepturine adults, DNA sequences of 532 bps were used for phylogenetic analyses. The analyses revealed that all larvae formed a clade with *L*. *o*. *ochraceofasciata* (Fig 3). Thus, they were concluded to be *L*. *o*. *ochraceofasciata*.

### Fungus-storing organs of larvae

All larvae (weight: 125.9 ± 66.2 mg, range = 73.7 to 227.0 mg, *n* = 5) molecularly identified as *L*. *o*. *ochraceofasciata* had cyst-like mycetomes girdling the outer surface of the basal part of the midgut adjacent to the foregut (*n* = 5) (Fig 1F and 1G, S1 Table).

### Behaviors of hatched larvae

We observed that the larvae fed on eggshells during and immediately after hatching (*n* = 23) (Fig 1H).

### Yeasts isolated from mycetangia, mycetomes, and eggshells

When microbial isolation was conducted from mycetangia of female adults, a single morphotype of fungi grew on the PDA plates. It was uniform whitish, round, and had a yeast-like colony morphology (Fig 1D). For the 26S gene, all DNA sequences of the yeasts (591 bps) were identical to each other within an individual and among individual beetles and showed 100% identity to *Scheffersomyces insectosa* (CBS 4287: GenBank KY109566) (mean ± SD = 1.3 × 10^5^ ± 1.7 × 10^5^ CFU/mycetangium, range = 8.8 × 10^2^ to 5.9 × 10^5^ CFU/mycetangium, *n* = 12) (S1 Table). For the ITS/5.8S gene, the DNA sequences of 12 representative isolates (617 bps) showed 99.7–100% identity to each other among individuals and 99.7–100% identity to *Sc*. *insectosa* (ATCC 66604: HQ652071). For the TEF gene, DNA sequences (663–782 bps) showed 98.6–99.9% identity to each other among individuals. Those of 9 out of 12 representative isolates (663–782 bps) were closest to *Sc*. *insectosa* (ATCC 66611: KC507457; 99.2–99.7% identity) and those of the remaining 3 isolates (744–782 bps) were closest to *Sc*. *parashehatae* (ATCC 58780: KC507459; 99.2–99.7% identity), followed by *Sc*. *insectosa* (ATCC 66611: KC507457; 99.0–99.5% identity).

Yeasts isolated from mycetomes of larvae showed a single morphotype (1.0 × 10^4^ ± 2.0 × 10^4^ CFU/mycetome, range = 1.2 × 10^2^ to 4.6 × 10^4^ CFU/mycetome, *n* = 5) that was the same as those from mycetangia of adults. The DNA sequences of 26S, ITS/5.8S, and TEF genes revealed that they were closest to the yeasts isolated from mycetangia of adults (S1 Table).

The same morphological trait of yeasts was isolated from the egg surface. The DNA sequences of 26S, ITS/5.8S, and TEF genes revealed that all three eggs contained the yeasts (3.5 × 10^3^ ± 1.6 × 10^3^ CFU/egg, range = 2.5 × 10^3^ to 5.3 × 10^3^ CFU/egg, *n* = 3) that were closest to the mycetangial yeasts (S1 Table). In two of the three eggs, in addition, another yeast that was closest to *Meyerozyma caribbica* (CBS 9966: MH545919; 100% identity for 26S) was detected (2.6 × 10^3^ ± 1.5 × 10^2^ CFU/egg, range = 2.5 × 10^3^ to 2.7 × 10^3^ CFU/egg, *n* = 2) (S1 Table).

**Fig 4:**
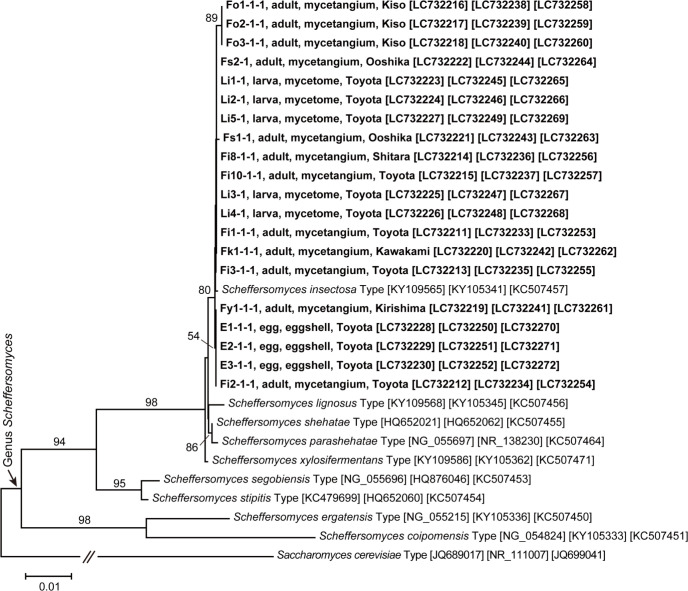
Phylogenetic placement of yeasts constantly-isolated from *Leptura ochraceofasciata*. A neighbor-joining phylogenetic tree was constructed using the DNA sequences of 26S (570 bps), ITS/5.8S (618 bps), and TEF (584 bps) genes. Bootstrap values (1000 replicates) of 50% or higher are shown at the nodes. For each yeast strain obtained from *L*. *ochraceofasciata* in this study, the name of the strain, developmental stage and isolation source of the host insect, and collection locality are indicated in bold. Sequence accession numbers are shown in brackets.

Due to multiple alignments of the DNA sequences of the studied and reference yeasts, 570 bps (26S), 618 bps (ITS/5.8S), and 584 bps (TEF) were used for the phylogenetic analyses. The analyses revealed that the isolated *Scheffersomyces* yeasts were conspecific with *Sc*. *insectosa* in the *Scheffersomyces* clade (Fig 4).

### Carbon assimilation test

The turbidity (OD_600_) of solution of the yeast associate showed a significant positive correlation with CFU (*y* = 1.30 × 10^5^ + 2.73 × 10^6^ *x*, *P* = 0.007).

To determine the assimilation of wood-associated carbon sources by the yeast symbiont, 16 different carbon sources including a treatment with no carbon source were tested using liquid media. When the yeast was cultured with no carbon source for 7 days, the turbidity increase was 0.00. The yeast assimilated corn-xylan very strongly and glucose, galactose, mannose, xylose, rhamnose, fructose, sucrose, cellobiose, and beech-xylan strongly (Table 4). Arabinose, galacturonic acid, glucuronic acid, mannan and carboxymethyl cellulose were not assimilated (Table 4).

**Table 4:** Growth of *Scheffersomyces insectosa* (strain: Fo1-1-1), the yeast symbiont of *Leptura ochraceofasciata* on different carbon sources.

Carbon source	ΔOD_600_^a^	Growth
d-Glucose	0.60	++
d-Galactose	0.50	++
d-Mannose	0.60	++
d-Xylose	0.55	++
l-Arabinose	0.02	–
l-Rhamnose	0.53	++
d-Fructose	0.66	++
d-Galacturonic acid	0.03	–
d-Glucuronic acid	0.02	–
Sucrose	0.67	++
Cellobiose	0.67	++
Xylan (corn)	1.27	+++
Xylan (beech)	0.82	++
Mannan	0.02	–
Carboxymethyl cellulose	0.01	–

–, no growth; +, moderate growth; ++, strong growth; +++, very strong growth.

^a^ Difference of the turbidity increase between culture media containing no and a given carbon source.

## Discussion

All female adults of *L*. *ochraceofasciata* obtained from multiple locations including two subspecies (*L*. *o*. *ochraceofasciata* and *L*. *o*. *ochrotela*) harbored only one species of yeast, *Sc*. *insectosa* in their mycetangia. All larvae of *L*. *ochraceofasciata* had mycetomes and harbored *Sc*. *insectosa* exclusively in them. *Scheffersomyces insectosa* was also recovered from all eggs examined. *Scheffersomyces insectosa* assimilated wood-associated sugars, including xylose, cellobiose, and xylan in culture. These results strongly suggest an intimate association between *L*. *ochraceofasciata* and *Sc*. *insectosa* through the insect’s life history. *Scheffersomyces insectosa* may benefit from the vectoring activity of *L*. *ochraceofasciata* from wood to wood. Given that only larvae of *L*. *ochraceofasciata* consume wood in its developmental stages, *Sc*. *insectosa* may help the larvae to digest wood. This is the first reported example of a lepturine-yeast association in Asia.

In mycetangia-bearing insects, there are many types of mycetangia, such as those showing differences in shape (pit, sac, or setal-brush) and presence/absence of secretion glands [10, 36]. Mycetangium sensu stricto represents a glandular type only, while mycetangium sensu lato includes a nonglandular one. In the present study, the type of mycetangia of *L*. *ochraceofasciata* was undetermined. Thus, it is tentatively used in a broad sense.

The 3D structure of ovipositor-associated mycetangia reconstructed based on micro-CT images revealed that the tube-like mycetangia are bendable in the beetle’s body. During oviposition, a female insect exposes her ovipositor to insert it into crevices of oviposition substrate and retract it after egg deposition (MK, FM, WT personal observation). The muscle bundle connected between the apex of the mycetangium and sclerotized spiculum ventrale of sternum VIII (Figs 1B and 2, S2 Movie). Thus, it is considered that a mycetangium is usually S-shaped inside the body and that it is tensioned when the ovipositor is protracted, resulting in the secretion of yeast cells. Symbiont-loading from mycetangia onto the egg surface may be synchronized with movement of the ovipositor for egg deposition.

In two European lepturines, *Oxymirus cursor* (Linnaeus) and *R*. *mordax*, their yeast symbionts are transmitted from mother to offspring via the surface of eggs: hatched larvae acquire yeast symbionts by ingesting the eggshells on which the yeast symbionts are present [16, 17]. In *L*. *ochraceofasciata*, similarly, the hatched larvae feed on their yeast-present eggshells. Vertical transmission mechanism of yeast symbionts may have been conserved in Lepturinae.

Interestingly, *Sc*. *insectosa* was isolated from mycetangia of one female before she emerged from her pupal chamber and two females immediately after they emerged from the natal wood. This repeated isolation of *Sc*. *insectosa* from newly eclosed adults suggests that female adults of *L*. *ochraceofasciata* incorporate *Sc*. *insectosa* into their mycetangia within pupal chambers. Meanwhile, budding yeast cells were present within mycetangia of female adults that were collected on flowers (Fig 2C). It is likely that *Sc*. *insectosa* reproduces within mycetangia after the female adults emerge from the natal wood.

In the carbon assimilation test, *Sc*. *insectosa* assimilated various sugars including wood-associated mono-, di-, and polysaccharides. Particularly, this yeast showed marked ability to assimilate corn- and beech-xylans. These data must be interpreted with caution, however, because the concentrations of these xylans were higher than the other carbon sources. This high concentration of xylans would cause relatively strong growth of the yeast. Nevertheless, the *L*. *ochraceofasciata*-associated *Sc*. *insectosa* evidently assimilates xylans and the degree of assimilation ability may vary among xylans. In contrast, *Sc*. *insectosa* (strain: SICYLG3) isolated from the gut of larvae of *Sinodendron cylindricum* (Linnaeus) (Lucanidae) assimilates xylan weakly [37]. Larvae of *Si*. *cylindricum* inhabit decayed wood [37], while those of *L*. *ochraceofasciata* can utilize physically hard wood (FM, WT personal observation), in which hemicelluloses including xylan are likely to exist abundantly. This physiological difference between yeast strains may be related to the microhabitats of their insect hosts.

Female adults and larvae of *L*. *ochraceofasciata* harbored *Sc*. *insectosa* exclusively in their fungus-storing organs, whereas another yeast, *Meyerozyma caribbica*-like yeast was detected from eggs together with *Sc*. *insectosa*. Given that *M*. *caribbica* is found widely in various artificial and natural environments [19] and that the oviposition experiment in this study was conducted under unsterilized conditions, it is likely that the presence of *Meyerozyma* sp. on eggs is due to contamination during the experiment.

*Leptura ochraceofasciata* constantly and exclusively possessed *Sc*. *insectosa* with marked abundance, whereas the yeast has been isolated from other xylophagous beetles: mycetangia of a female adult of the European lepturine, *Sti*. *maculicornis* (DeGeer) (formerly, *L*. *maculicornis*) [18, 38], and the gut of larvae of the lucanid *Si*. *cylindricum* obtained from decayed wood of a beech tree in Switzerland [37]. These suggest an asymmetrical interdependence between *L*. *ochraceofasciata* and *Sc*. *insectosa*. *Leptura ochraceofasciata* might obligatorily depend on *Sc*. *insectosa*, but *Sc*. *insectosa* might facultatively depend on *L*. *ochraceofasciata*. Alternatively, at a local scale, *Sc*. *insectosa* might obligatorily depend on *L*. *ochraceofasciata*. In Japan, *L*. *ochraceofasciata* is one of the most common lepturines in primary and secondary forests from low to high elevation areas [21]. On the other hand, *Sc*. *insectosa* is a rare yeast and has been isolated from xylophagous beetles twice in Europe [18, 37, 38]. Further study is required to determine to what extent *Sc*. *insectosa* depends on *L*. *ochraceofasciata* by examining many wood-inhabiting insects living sympatrically with *L*. *ochraceofasciata*.

In Lepturinae, two lineages of yeasts (i.e., *H*. *rhagii* and *Scheffersomyces* spp.) have been primarily known as mycetangium-associated and/or mycetome-associated yeasts: *H*. *rhagii* from *R*. *inquisitor* and *Carilia virginea* (Linnaeus) (formerly, *Gaurotes virginea*), *Sc*. *insectosa* from *Sti*. *maculicornis* and *L*. *ochraceofasciata*, *Sc*. *shehatae* from *Pachytodes cerambyciformis* (Schrank) (formerly, *L*. *cerambyciformis*), *Sc*. *stipitis*-related yeast from *Sti*. *rubra*, *Scheffersomyces* sp. from *R*. *bifasciatum* Fabricius, *R*. *mordax*, and *R*. *sycophanta* (Schrank) [5, 16–19]. In addition, *Candida* sp. was isolated from mycetangia of *Anastrangalia sanguinolenta* (Linnaeus) (formerly, *L*. *sanguinolenta*) [18]. Many elongated-mycetangium-bearing lepturine species including *L*. *quadrifasciata* Linnaeus (formerly, *Stenura quadrifasciata*) harbor unidentified yeasts in mycetangia [17]. Although most of these findings are based on small sample numbers of living or dried insect specimens [17, 18], it is suggested that the association with yeasts may be ubiquitous in Lepturinae. Further studies examining large numbers of individuals and species comprehensively using molecular techniques will elucidate the evolutionary process of lepturine-yeast symbioses.

## Supporting information

S1 Movie Micro-CT sections of female genitalia of *Leptura ochraceofasciata*. (MP4)

S2 Movie 3D image of female genitalia of *Leptura ochraceofasciata* based on micro-CT. (MP4)

S1 Table *Leptura ochraceofasciata* used for yeast isolation and the isolated yeasts. (PDF)
