# Supplementary material for: Yeast associated with flower longicorn beetle Leptura ochraceofasciata (Cerambycidae: Lepturinae), with implication for its function in symbiosis
Source: PLoS One. 2023 Mar 22;18(3):e0282351. doi: 10.1371/journal.pone.0282351 (PMC10032543; doi:10.1371/journal.pone.0282351)
Supplement: S1 Table — (PDF) [file pone.0282351.s003.pdf]

S1 Table. ***Leptura ochraceofasciata*** **used for yeast isolation and the isolated yeasts.**

Beetle ID	Developmental stage	Body length (mm)	Elytral length (A) (mm)	Body weight (mg)	Mycetangial length (B) (mm)	Relative mycetangial length (B/A)	Yeast		
							Isolation source	Species	CFU/isolation source
Fi1	Adult	16.65	11.79	161.0	2.99	0.25	Mycetangium	*S. insectosa*	8.8×10^2
Fi2	Adult	16.20	11.73	156.6	2.57	0.22	Mycetangium	*S. insectosa*	1.8×10^4
Fi3	Adult	16.35	11.58	158.5	3.09	0.27	Mycetangium	*S. insectosa*	5.9×10^5
Fi8	Adult	19.98	13.15	247.7	2.85	0.22	Mycetangium	*S. insectosa*	2.7×10^5
Fi10	Adult	18.88	12.92	227.8	2.99	0.23	Mycetangium	*S. insectosa*	1.0×10^5
Fo1	Adult	18.18	12.85	217.6	2.96	0.23	Mycetangium	*S. insectosa*	1.7×10^5
Fo2	Adult	17.64	12.65	209.8	2.62	0.21	Mycetangium	*S. insectosa*	4.5×10^3
Fo3	Adult	17.15	12.19	198.2	2.79	0.23	Mycetangium	*S. insectosa*	6.0×10^4
Fk1	Adult	18.70	12.69	214.4	2.80	0.22	Mycetangium	*S. insectosa*	6.0×10^3
Fs1	Adult	17.77	12.43	188.8	n.a.	n.a.	Mycetangium	*S. insectosa*	1.7×10^5
Fs2	Adult	18.49	13.41	215.7	n.a.	n.a.	Mycetangium	*S. insectosa*	2.7×10^3
Fy1	Adult	16.44	10.46	115.2	2.85	0.27	Mycetangium	*S. insectosa*	1.4×10^5
Li1	Larva	n.a.	n.a.	158.9	n.a.	n.a.	Mycetome	*S. insectosa*	6.1×10^2
Li2	Larva	n.a.	n.a.	227.0	n.a.	n.a.	Mycetome	*S. insectosa*	1.6×10^3
Li3	Larva	n.a.	n.a.	73.7	n.a.	n.a.	Mycetome	*S. insectosa*	1.2×10^2
Li4	Larva	n.a.	n.a.	76.6	n.a.	n.a.	Mycetome	*S. insectosa*	1.9×10^3
Li5	Larva	n.a.	n.a.	93.1	n.a.	n.a.	Mycetome	*S. insectosa*	4.6×10^4
E1	Egg	n.a.	n.a.	n.a.	n.a.	n.a.	Eggshell	*S. insectosa*	2.5×10^3
								Meyerozyma sp.	2.5×10^3
E2	Egg	n.a.	n.a.	n.a.	n.a.	n.a.	Eggshell	*S. insectosa*	5.3×10^3
E3	Egg	n.a.	n.a.	n.a.	n.a.	n.a.	Eggshell	*S. insectosa*	2.7×10^3
								Meyerozyma sp.	2.7×10^3

n.a., not applicable.
